# Bufalin Inhibits Proliferation and Induces Apoptosis in Osteosarcoma Cells by Downregulating MicroRNA-221

**DOI:** 10.1155/2016/7319464

**Published:** 2016-12-15

**Authors:** Jianjun Zhang, Jingjing Sha, Yan Zhou, Kun Han, Yaling Wang, Yang Su, Xiuyi Yin, Haiyan Hu, Yang Yao

**Affiliations:** ^1^Oncology Department of Shanghai Jiao Tong University Affiliated Sixth People's Hospital, Shanghai 200233, China; ^2^Jin Yang Community Health Service Center, Shanghai 200136, China

## Abstract

Bufalin, a major component of the Chinese medicine ChanSu, which is prepared from the skin and parotid venom glands of toads, has shown cytotoxicity in several malignant tumors. Here, we reported that bufalin inhibited proliferation and induced mitochondria-dependent apoptosis in U-2OS and Saos-2 osteosarcoma cells with intracellular reactive oxygen species (ROS) production. By microRNA (miR) array analysis and quantitative reverse transcription polymerase chain reaction, we found that miR-221 was downregulated after treatment with bufalin. In accordance with TargetScan prediction and luciferase reporter assay, Bcl2 binding component 3 (BBC3) was the direct target of miR-221. Furthermore, upregulating miR-221 by its MIMIC and suppressing BBC3 by small interfering RNA (siRNA) reversed the effects of bufalin on osteosarcoma cells. Collectively, our data indicate that bufalin inhibits cell proliferation and induces mitochondria-dependent apoptosis in osteosarcoma cells through downregulating miR-221 and triggering BBC3 expression.

## 1. Introduction

Osteosarcoma (OS) is the most common primary sarcoma of bone in children and adolescents. The 5-year survival of patients with OS has improved considerably from 20% to 65% since the 1980s with the advent of multiagent chemotherapy [1, 2]. But after high-dose, frequent neoadjuvant, and adjuvant chemotherapy, numerous patients with OS become multidrug resistant. The prognosis for those with recurrent or metastatic diseases remains very poor [3]. Thus, it is urgently needed to find alternative anticancer agents that will avoid chemoresistance and improve clinical outcomes. The skin and parotid venom glands of toads have been widely used for centuries in traditional Chinese medicine for the treatment of various ailments such as heart failure, hypertension, cancer, and sores [4–6]. Bufalin is the major component of the Chinese medicine ChanSu, which is obtained from the skin and parotid venom glands of toads. It has been shown to increase vascular resistance and blood pressure [7, 8]. In the past decades, more and more researches have focused on its anticancer effects [9, 10]. However, the effects of bufalin on OS remain poorly understood. In the present study, in order to identify the possible network activated by bufalin in OS cells, we conducted microRNA (miR) array analysis in U-2OS and Saos-2 cells after treatment with bufalin. Our findings suggested that bufalin activated the mitochondria-dependent apoptosis via downregulation of miR-221.

## 2. Materials and Methods

### 2.1. Cells Culture and Proliferation Assay

Human U-2OS and Saos-2 OS cells were purchased from Shanghai Institute of Biochemistry and Cell biology. The cell lines were maintained in DMEM (Invitrogen, Carlsbad, CA, USA) supplemented with 10% fetal bovine serum (FBS, Hyclone, Logan, UT, USA) at 37°C in an incubator with a constant air flow of 5% CO_2_ and 95% O_2_ and routinely passaged at 2-3-day intervals. Bufalin (Sigma-Aldrich, St Louis, MO, USA) was dissolved to 50 mM in dimethyl sulfoxide (Sigma-Aldrich, St. Louis, MO, USA) and stored at −80°C. The final concentrations were 0.05–10 *μ*M. As for reverse test, once the cells were 80% confluent, they were starved in DMEM with 1% FBS for 24 h and then transfected with miR-221-MIMIC (oligo-deoxynucleotides, Shanghai GenePharma Company, Shanghai, China), NC control, or small interfering RNA- (siRNA-) Bcl2 binding component 3 (BBC3) using Lipofectamine 2000 reagent (Invitrogen, Carlsbad, CA, USA), respectively. After transfection, the cells were treated with bufalin for 24 h. Cell Counting Kit-8 (CCK-8, Dojindo Molecular Technologies, Dojindo, Japan) was used to perform cell proliferation assay. Absorbance value was measured for each well at a wavelength of 450 nm, with the reference wavelength set at 630 nm. Data were calculated from three independent experiments, and the percentage of bufalin-induced cell growth inhibition was determined by comparison to untreated control cells.

### 2.2. Evaluation of Cell Apoptosis

After transfection with miR-221-MIMIC or siRNA-BBC3 followed by treatment with bufalin for 24 h, the OS cells were harvested and stained with Annexin V-fluorescein isothiocyanate (FITC) and propidium iodide (PI) apoptosis detection kit (Kaiji Bio-tech, Nanjing, China). The resulting fluorescence was measured by fluorescence-activated cell sorting scan (FACS) flow cytometry (Becton Dickinson, Mountain View, CA, USA).

### 2.3. Measurement of Intracellular Reactive Oxygen Species (ROS)

The intracellular ROS was measured in OS cells (2 × 10^6^ per well) grown for 24 h in 6-well plates (Corning, USA) in the same condition as proliferation assay. After being digested by 2.5% trypsin enzyme, the cells were harvested and washed with PBS for twice and then loaded with 10 *μ*M 2′,7′-dichlorodihydrofluorescein diacetate (H2DCFDA) (Invitrogen, Carlsbad, CA, USA) for 25 min in the dark. Afterwards, cells were washed with PBS. A FACS flow cytometer was used to measure the resulting fluorescence. Untreated control cells were used as negative control.

### 2.4. Mitochondrial Membrane Potential (ΔΨm) Assay

The ΔΨm was evaluated using the 5,5′,6,6′-tetrachloro-1,1′,3,3′-tetraethyl-imidacarbocyanine iodide (JC-1, Beyotime, Jiangsu, China) dye according to the manufacturer's instructions. Briefly, both U-2OS and Saos-2 cells (1 × 10^5^ per well) were seeded in 4-well tissue-culture slides in the same condition as proliferation assay. After 24 h, cells were washed with PBS and stained with JC-1 for 30 min at 37°C in the dark. Stained cells were harvested, washed, and subjected to analysis by a confocal microscopy (LSM710, Zeiss, Germany). The different colors for fluorescence of JC-1 represented different ΔΨm.

### 2.5. Detection of Differentially Expressed miRNAs by miRNA Microarray

The Saos-2 cells treated with or without bufalin at its half maximal inhibitory concentration (IC50) for 24 h were harvested and then analyzed using miRNA microarray (Kangcheng Biotech Company, Shanghai, China). Briefly, total RNA was isolated by Trizol reagent (Invitrogen, Carlsbad, CA, USA) according to the user's guide. The miRNA was separated from 30–50 mg of the total RNA, labeled with the miRCURY Hy3TM/Hy5TM Power Labeling Kit (Exiqon, Vedbaek, Denmark), and hybridized to a miRCURY LNA Array (Exiqon, v11.0). An Axon GenePix 4000B microarray scanner (Axon Instruments Inc, Union City, CA, USA) was used for scanning and GenePix Pro v6.0 was used to read the raw image intensity. Unsupervised hierarchical clustering was performed on the miRNA expression profile. Each miRNA present in the database was mapped to a precise location in the human genome using a Basic Local Alignment Search Tool (BLAST) with the default parameters and the maps available from the National Center for Biotechnology Information Human Genome Resources (https://www.ncbi.nlm.nih.gov/). In order to validate the present study data, the clones corresponding to each miRNA were identified and mapped to the human genome.

### 2.6. Targeted In Vitro Luciferase Reporter Assay

The psi-Check2 plasmid (Promega Corporation, Fitchburg, WI, USA) was used for the expression of the miR-221 target BBC3 3′-untranslated region (UTR). The wild-type (WT) Check2-BBC3 construct contained one miR-221 response element from BBC3 3′-UTR, and the mutant (MUT) Check2-BBC3 construct had two nucleotides replaced within the seed sequence. The sequences used to create the Check2-BBC3 were as follows: forward primer 5′-**AACTCGAG** TGCACTGACGGAGATGCG-3′ (XhoI site in bold) and reverse primer 5′-**AATGCGGCCGC** CACTGTTCCAATCTGATTTTAT-3′ (NotI site in bold). The oligonucleotides were annealed and inserted into the psi-Check2 vector. Site-directed mutagenesis was performed using Quick Change kit (Stratagene, USA). Mutation was done by replacement of the predicted miR-221 binding site AUGUAGC to AAGAAGC. The empty vector (psi-Check2) was used as a negative control (NC). HEK-293 cells were transfected with 0.2 *μ*g of the reporter plasmid and 0.01 *μ*g of the psi-Check2 control plasmid and 100 nM miR-221-MIMIC (miR-221 precursor) and the NC miRNA per well on 96-well plates. After incubation for 24 h, cells were subjected to a luciferase reporter assay using the Luciferase Assay System (Promega Corporation, Fitchburg, WI, USA). The Renilla luciferase values were normalized to firefly, and the ratio of Renilla/firefly values was presented. Data were collected from 3 separate experiments.

### 2.7. Quantitative Real-Time PCR of miR-221 and BBC3 Expression

U-2OS and Saos-2 cells were transfected with miR-221-MIMIC (100 nM) using the Lipofectamine 2000 reagent. The process of RNA isolation was followed as mentioned above. U6 was used as the internal control. The miR-221 and U6 levels were measured using the miRNAs RT-PCR Quantitation Kit (Shanghai GenePharma Company, Shanghai, China). The BBC3 primers were as follows: forward 5′-AGAGGGAGGAGTCTGGGAGTG-3′ and reverse 5′-GCAGCGCATATACAGTATCTTACAGG-3′. The results were normalized to GAPDH using the following primers: forward 5′-GAAGGTGAAGGTCGGAGT-3′ and reverse primer 5′-GAAGATGGTGATGGGATTTC-3′. Quantitative reverse transcription polymerase chain reaction (qRT-PCR) for BBC3 was performed using the SYBR Green Master Mix Kit (Takara, Japan). The fold changes for miR-221 and BBC3 expression were calculated through the relative quantification using 2^−ΔΔCt^. All the reactions were performed in triplicate and repeated thrice.

### 2.8. Western Blot Analysis

The OS cells were lysed in RIPA buffer in the presence of proteinase inhibitor cocktail (Shanghai Shenergy Biocolor BioScience & Technology Company, Shanghai, China). Protein concentration was determined using BCA method (Bios, Beijing, China). Aliquots (25 *μ*g) were separated on 10% SDS-PAGE and transferred to nitrocellulose membranes. Afterwards, the membranes were probed with a primary antibody against BBC3 (mouse monoclonal, Cell Signaling Technology, MA) and then incubated with the appropriate horseradish peroxide-conjugated secondary antibody at 1 : 1000 dilution. The intensity of the protein fragments was displayed with an X-ray image film-processing machine (Kodak, Rochester, NY, USA).

### 2.9. Knocking down BBC3 with siRNA

Specific siRNA targeting to BBC3 were sense 5′-UCUCAUCAUGGGACUCCUGTT-3′ and antisense 5′-UUGAGGUCGUCCGCCAUCCTT-3′, which were purchased from Jima Biocom (Shanghai, China). Then they were transfected into U-2OS and Saos-2 cells to suppress the function of BBC3 using Lipofectamine 2000.

### 2.10. Statistical Analysis

The data analysis was performed using commercially available software (Statistical Package for the Social Sciences version 15.0, SPSS, Inc., Chicago, Illinois, USA). The data were presented with mean ± SD. One-way analysis of variance was used to test the differences between the mean value. A *P* value < 0.05 was considered to be statistically significant.

## 3. Results

### 3.1. Bufalin Activated Mitochondrial Apoptotic Pathway with Intracellular ROS Production

**Figure 1:**
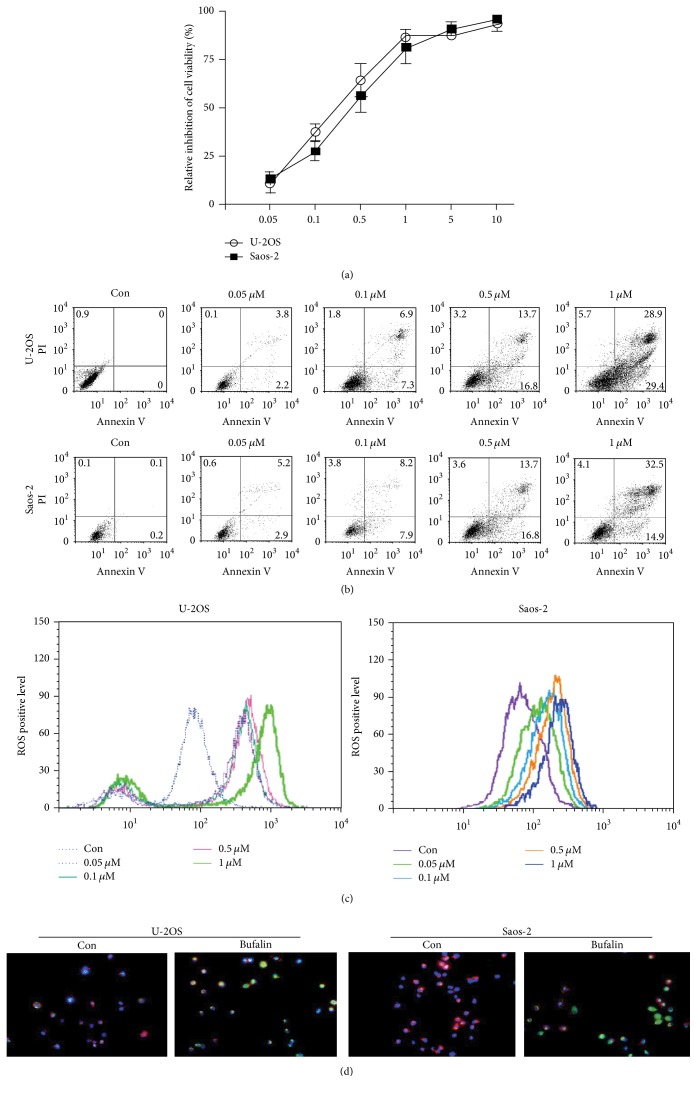
Bufalin regulates apoptosis, ROS production, and mitochondrial membrane potential in OS cells. (a) After treatment with various concentrations (0.05–10 *μ*M) of bufalin for 24 h, viability of U-2OS and Saos-2 cells was determined using the CCK-8 assay. The inhibition ratio of cell viability increased in a dose-dependent manner. (b) U-2OS and Saos-2 cells were treated with 0.05–1 *μ*M of bufalin and stained with Annexin V/PI. The apoptotic cells were detected by FCM. The apoptosis ratio changed in a dose-dependent manner. (c) After exposure to bufalin in different concentrations, fluorescence intensity indicating the intracellular ROS production was evaluated by FCM. ROS production increased gradually as bufalin concentration rose. (d) The ΔΨm of OS cells was measured using the JC-1 dye. After being treated with IC50 bufalin, the OS cells exhibited a reduction of ΔΨm as demonstrated by a decrease in red/green ratio. The red/green ratio in the control and bufalin group was 57.26 ± 7.13% versus 5.37 ± 0.63% for U-2OS cell, respectively (*P* < 0.01), while it was 60.63 ± 5.67% versus 3.83 ± 0.33% for Saos-2 cell (*P* < 0.01).

The concentration range of bufalin in this experiment was 0.05–10 *μ*M. As shown in Figure 1(a), bufalin significantly inhibited cell proliferation of U-2OS and Saos-2 cells in a dose-dependent manner. The IC50 at 24 h was calculated and a weighted average of three independent results were taken, and the final IC50 of bufalin on U-2OS and Saos-2 cells was 0.297 and 0.318 *μ*M, respectively. To explore the mechanism by which bufalin caused viability loss in OS cells, apoptosis experiments were carried out. In line with the proliferation inhibition effect, bufalin treatment also induced apoptosis of OS cells in a dose-dependent manner (Figure 1(b)). The production of intracellular ROS plays a critical role in the proapoptotic activities of numerous anticancer agents. Bufalin caused a concentration-dependent increase of ROS production in U-2OS and Saos-2 cells (Figure 1(c)). Depolarization of the ΔΨm has been suggested to be central to the intrinsic apoptotic pathway, which is characterized by the permeabilization of the mitochondrial outer membrane. Here we also detected whether bufalin affected ΔΨm using the fluorescent dye JC-1. A dissipation in ΔΨm was associated with a decrease in the red/green fluorescent ratio. The OS cells exposed to IC50 bufalin exhibited a reduction of ΔΨm (Figure 1(d)).

### 3.2. Bufalin Downregulated the Expression of miR-221

**Figure 2:**
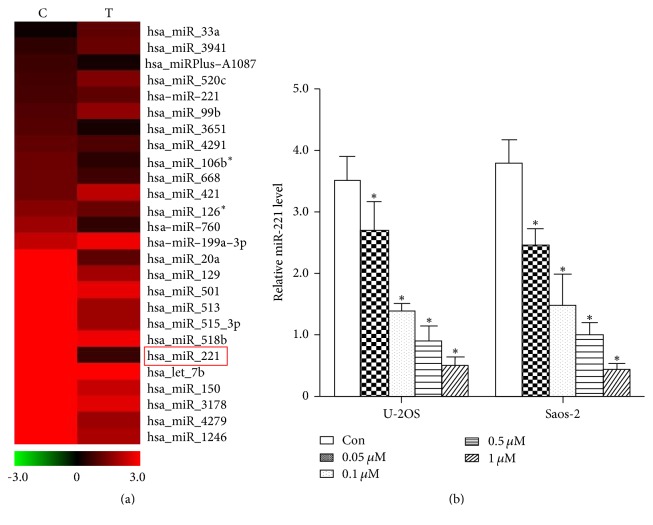
Bufalin downregulates miR-221 in U-2OS and Saos-2 cells. (a) Microarray analysis was used to compare the expression profiles of miRNAs in Saos-2 cells that were untreated or treated with bufalin at its IC50. miR-221, one of the most markedly downregulated miRNAs, was labeled with a red box. (b) Detected by qRT-PCR, miR-221 level dramatically decreased 2–5-fold after being treated with bufalin in both U-2OS and Saos-2 cells (^*∗*^ *P* < 0.01 compared with the untreated control group).

In order to explore the mechanism by which bufalin inhibits the proliferation of OS cells, we screened the miRNAs that were modulated by bufalin in Saos-2 cells using miRNA microarray. miR-221 was one of the markedly downregulated miRNAs, which provokes our interest due to its tumor suppressor role in many other cancer cells (Figure 2(a)). The effect of bufalin in downregulating miR-221 was confirmed using qRT-PCR in both U-2OS and Saos-2 cells. As shown in Figure 2(b), bufalin downregulated miR-221 in a dose-dependent manner.

### 3.3. BBC3 Was the Target of miR-221

**Figure 3:**
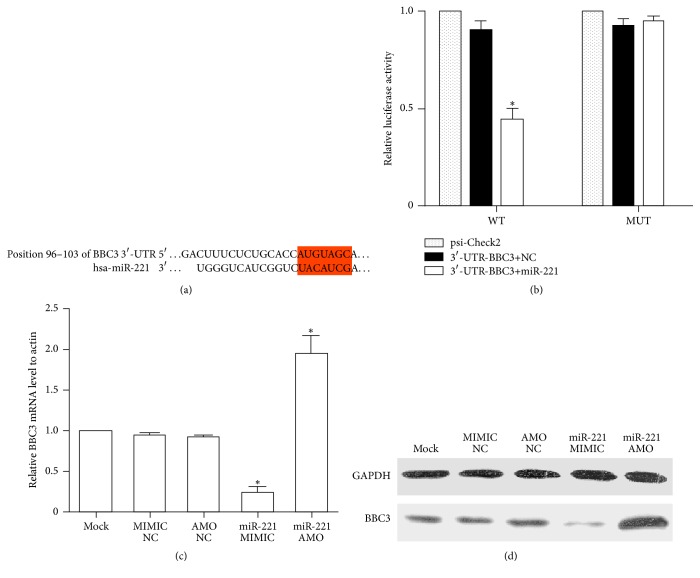
Functional screening of miR-221 target sites using luciferase reporter assay. (a) The selection criteria of the miRNA targets were based on their common detection in the target prediction online databases (TargetScan7.0 http://www.targetscan.org/) as well as the full complementarity between the seed region of miR-221 and 3′-UTR of BBC3. (b) HEK 293 cells cotransfected with miR-221-MIMIC, psi-Check2, psi-Check2-BBC3, or MUT-psi-Check2-BBC3. The luciferase activity levels were measured 24 h after transfection; results from at least three separate experiments are presented as means ± SE. The results show the regulation of BBC3 by miR-221 (^*∗*^ *P* < 0.01 compared with the control). (c) After transfection for 24 h, the BBC3 mRNA expression levels in Saos-2 cells transfected with miR-221-MIMIC and miR-221-AMO were determined by qRT-PCR (^*∗*^ *P* < 0.01 compared with the control). (d) The BBC3 protein expression levels in Saos-2 cells transfected with miR-221-MIMIC and miR-221-AMO were analyzed by Western blot at the same time to qRT-PCR.

The potential targets of miR-221 were prognosed by TargetScan analysis. There was one seed sequence for miR-221 in the 3′-UTR of BBC3, an important regulatory factor of cell apoptosis (Figure 3(a)). To test whether miR-221 directly interacts the 3′-UTR of BBC3, we cloned WT or MUT miR-221-BBC3 response element into the psi-Check2 plasmid downstream of the luciferase reporter. HEK293T cells were transfected with miR-221-MIMIC and BBC 3′-UTR vectors. As displayed in Figure 3(b), the reporter activity of WT but not MUT Check2-BBC3 inversely correlated with the miR-221 expression levels, it was approximately 35–40% of the control group (*P* < 0.001). To verify the regulatory effect miR-221 on BBC3 protein, we also transfected miR-221-AMO (anti-microRNA oligonucleotide, GenePharma Biotech Company, Shanghai, China), and miR-221-MIMIC into Saos-2 cells, then BBC3 was quantified by qRT-PCR (Figure 3(c)) and Western blot (Figure 3(d)). Either mRNA or protein levels of BBC3 were closely related to miR-221 levels.

### 3.4. Upregulating miR-221 and Suppressing BBC3 Reversed the Effects of Bufalin on OS Cells

**Figure 4:**
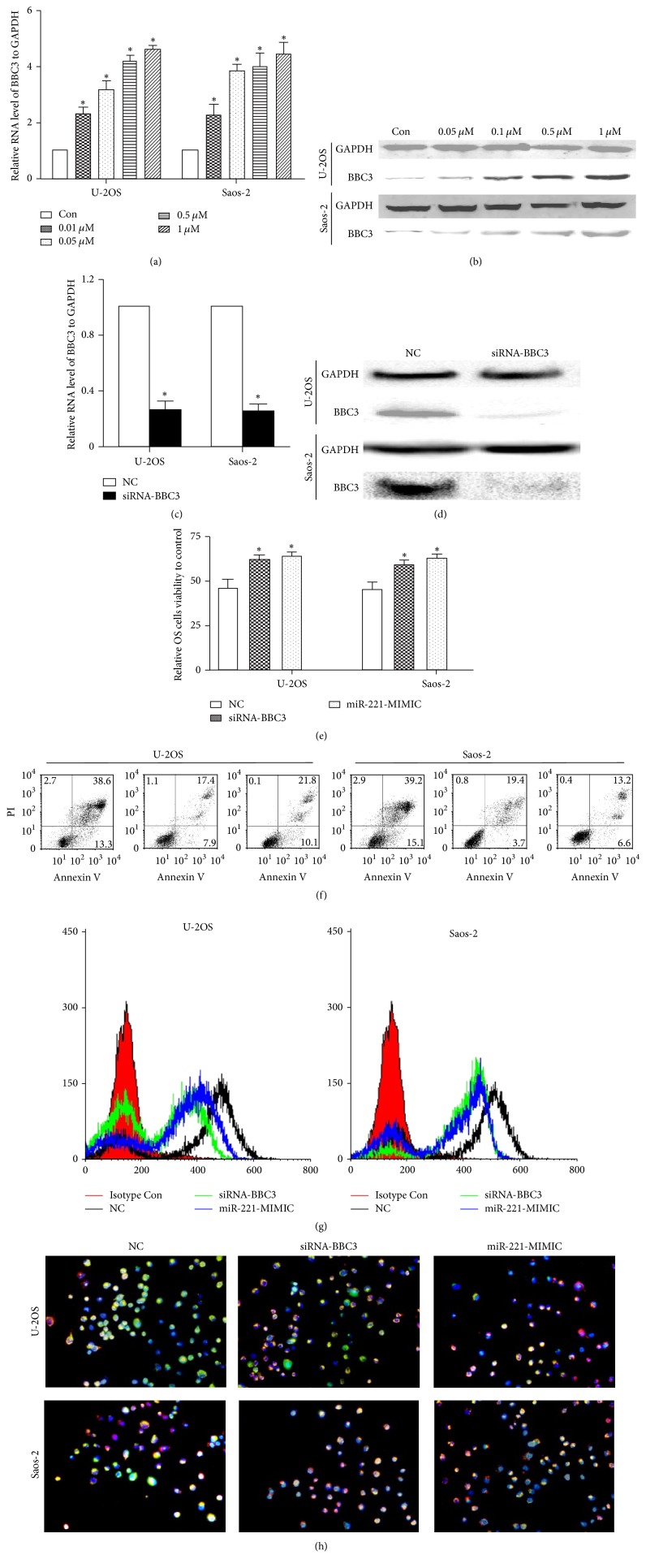
miR-221/BBC3 plays an important role in the effects of bufalin on OS cells. (a) After being treated with different concentrations of bufalin, the mRNA levels of BBC3 were upregulated in a dose-dependent manner (^*∗*^ *P* < 0.01 compared with the control). (b) The change of BBC3 protein level was in line with the tendency of mRNA. (c) The mRNA levels of BBC3 in U-2OS and Saos-2 cells decreased approximately 70% after being transfected with siRNA-BBC3 (^*∗*^ *P* < 0.01 compared with the control). (d) The protein levels of BBC3 had the same tendency toward mRNA. (e) miR-221-MIMIC or siRNA-BBC3 countered bufalin's effects on cell viability. After transfection, the OS cells were treated with IC50 bufalin for 24 h; according to the results of CCK-8 assay, the viability of U-2OS and Saos-2 cells transfected with miR-221-MIMIC or siRNA-BBC3 was significantly higher than in the NC group (^*∗*^ *P* < 0.01 compared with the control). (f) Changes in the apoptotic ratio of U-2OS and Saos-2 cells were detected by flow cytometry. The apoptotic ratio in the miR-221-MIMIC or siRNA-BBC3 group was markedly lower than in the NC group (^*∗*^ *P* < 0.01 compared with the control). (g) After being transfected with miR-221-MIMIC or siRNA-BBC3, intracellular ROS production in U-2OS and Saos-2 cells decreased markedly compared to the control group. (h) The ΔΨm in miR-221-MIMIC group or siRNA-BBC3 group was significantly higher than in the NC group. The red/green ratio in U-2OS cell transfected with miR-221-MIMIC or siRNA-BBC3 was 34.57 ± 6.26% and 31.27 ± 4.33%, comparatively lower than 61.67 ± 5.13% in the NC group (*P* < 0.01). In Saos-2 cell, the ratio was 33.33 ± 4.26% and 36.4 ± 5.63% versus 63.33 ± 7.67% (*P* < 0.01).

First of all, we clarified that bufalin significantly increased both the BBC3 mRNA and protein levels in U-2OS and Saos-2 cells in a dose-dependent manner (Figures 4(a) and 4(b)). Afterwards, we designed and synthesized siRNA-BBC3, which reduced mRNA and protein levels of BBC3 in OS cells significantly (Figures 4(c) and 4(d)). In order to know the role of miR-221 and BBC3 in the effects of bufalin on OS cells, U-2OS and Saos-2 cells were transfected with miR-221-MIMIC or siRNA-BBC3 or NC, respectively, and then treated with bufalin for 24 h; the viability of U-2OS and Saos-2 cells was detected by CCK-8 assay. As displayed in Figure 4(e), the miR-221-MIMIC or siRNA-BBC3 significantly countered bufalin's effects on cell viability, when compared with the NC group. Likewise, the apoptotic ratio of the miR-221-MIMIC or siRNA-BBC3 group was significantly decreased (Figure 4(f)). Furthermore, the effects of bufalin on ROS production and ΔΨm in OS cells were also reversed by miR-221-MIMIC or siRNA-BBC3 (Figures 4(g) and 4(h)).

## 4. Discussion

Neoadjuvant and adjuvant chemotherapy combined with complete surgical removal of all lesions is the main therapeutic strategy of OS. However, conventional chemotherapeutic approaches for OS patients may cause severe toxicity and deteriorated quality of life [11]. Poor physical fitness and severe toxicity which followed chemotherapy often become the main reasons for the patients to quit therapy. As a result, a considerable number of patients may develop recurrent and metastatic diseases. Thus, it is important to find alternative anticancer agents with low toxicity for OS. Traditional Chinese medicine with low side effects is gaining momentum in the treatment of a broad spectrum of tumors in recent years. Bufalin, a major effective component the Chinese medicine ChanSu, has demonstrated significant antitumor activity in several malignancies, such as breast, lung, and prostate cancer [12–15]. However, very few studies have been carried on the effects of bufalin on OS. In the present study, we found that bufalin caused viability loss and induced apoptosis in U-2OS and Saos-2 cells in a dose-dependent manner. Recently, much has been understood about the crude mechanism of bufalin's anticancer effects. Hashimoto et al. [16] reported bufalin specifically effected on the amounts of both topoisomerase (topo) II alpha and II beta and the activity of topo II. Pastor and Cortés [17] found bufalin affected the kinetics of DNA repair. Huang et al. [18] stated that bufalin arrested the G0/G1 stage in human bladder cancer cells through inhibiting cyclin D, cyclin E, cyclin-dependent kinase (CDK) 2, and CDK4. Sun found bufalin triggered ROS mediated apoptosis in lung cancer cells [19]. Mitochondria are widely believed to be the source of ROS [20]. ROS production has been demonstrated following the administration of many mitochondria-dependent apoptotic stimuli. Keeping ΔΨm is essential to preserve cell life. It is an important sign of apoptosis that ΔΨm collapses. In the present study, we also investigated the intracellular ROS production and ΔΨm in OS cells treated with bufalin using H2DCFDA and JC-1 dye. ΔΨm was calculated as the red/green fluorescent ratio. As shown in Figure 1(c), the florescence intensity of H2DCFDA increased progressively as bufalin concentration rose, but lower levels of red fluorescence and higher levels of green fluorescence were observed in the OS cells after exposure to bufalin (Figure 1(d)), indicating an increase of ROS production and a dissipation of ΔΨm in this process, respectively. Collectively, these results suggested that ROS-involved mitochondrial apoptotic pathway was triggered in bufalin-induced cell apoptosis.

miRNAs, typically 20–22 nucleotides in length, posttranscriptionally regulate the expression of target genes involved in many biologic processes, demonstrating far-reaching effects on the cellular biology of development and cancer. In recent years, emerging studies demonstrated that miRNAs such as miRNA-29 and miRNA-128 play an important role in the pathogenesis and progression of OS [21, 22]. Some researchers also investigated whether miRNAs took part in bufalin's effects on malignancies. Zhao et al. found that bufalin induced apoptosis in gastric cancer cell by downregulation of miR-298 [23]. Wang et al. reported that miR-155-5p was the key factor in the apoptotic effect of bufalin on triple-negative breast cancer cells [24]. miR-127-3p and miR-183 were suggested to be involved in bufalin's effects on ovarian cancer [25, 26]. As we know, the expression of miRNAs has tissue and time specificity. To further explore the exact mechanism of bufalin's effects on OS cells, we screened the miRNAs modulated by bufalin using miRNAs array. We identified miR-221 as one of the most downregulated miRNAs in Saos-2 Cells treated with bufalin. miR-221, located on the X chromosome, has been widely reported as oncogene and overexpressed in various kinds of cancer. Wang et al. reported that miR-221 was overexpressed and associated with poor survival in pancreatic ductal adenocarcinoma [27]. Liu et al.'s study showed that miR-221 promoted proliferation and migration of gastric cancer cell [28]. miR-221 was also suggested to be a poor potential biomarker for predicting the survival of breast cancer [29]. Many key factors of cell cycle and survival have proved to be the targets of miR-221, such as CDK inhibitory proteins p27Kip1 [30], proapoptotic BH3-only protein Bmf [31], and estrogen receptor alpha [32]. Here we predicted and verified that BBC3 was the direct target of miR-221. BBC3, also termed as p53 upregulated modulator of apoptosis (PUMA), plays an important role in suppressing tumor growth. It belongs to the Bcl-2 family of proteins responsible for maintaining mitochondrial outer membrane integrity by controlling the mitochondrial apoptotic pathway [33]. As a BH3-only protein, BBC3 interacts with antiapoptotic Bcl-2 and Bcl-X(L) and induces Bax-dependent apoptosis. In the present study, BBC3-3′-UTR and its mutated 3′-UTR dual luciferase reporter vectors (psi-Check2) were used to validate the negative regulation of BBC3 by miR-221, whose MIMIC (pre-miR-221) was transfected into HEK-293 cells using Lipofectamine 2000. Our results confirmed that there was strong correlation between miR-221 and BBC3. In order to further investigate whether miR-221/BBC3 plays an important role in the effects of bufalin on OS cells, the reverse test was performed. As expected, after transfecting miR-221-MIMIC or siRNA-BBC3 into U-2OS and Saos-2 cells, the effects of bufalin on cell proliferation and apoptosis, ROS production, and ΔΨm dissipation were reversed significantly.

As we know, apoptosis is a complicated systematic course and a lot of factors are involved in this process. Some molecules act as master regulators of the proapoptotic activity, while others served as secondary regulators. As mentioned above, miR-221 and its target BBC3 were suggested to be master regulators in the effects of bufalin on OS cells, but the apoptotic effect was not reversed completely by miR-221-MIMIC or siRNA-BBC3 (Figure 4(f)). In our opinion, this may be attributed to the potential involvement of other microRNAs including miR-3651, miR-106b, miR-668, miR-760, and miR-20a, which were also downregulated after treatment with bufalin (Figure 2(a)). However, they were all not downregulated as markedly as miR-221, indicating their potential involvement as secondary regulators.

In summary, our proof-of-concept study demonstrated that bufalin inhibited proliferation and induced mitochondria-dependent apoptosis in U-2OS and Saos-2 cells. Moreover, we identified BBC3 as a target of miR-221, which was downregulated in this process. Taken together, these findings provide a basic rationale for further assessment of bufalin as a promising candidate for the treatment of OS.
